# AI Scribes: Are We Measuring What Matters?

**DOI:** 10.2196/89337

**Published:** 2026-02-06

**Authors:** Enrico Coiera, David Fraile-Navarro

**Affiliations:** 1 Australian Institute of Health Innovation, Macquarie University, Level 6, 75 Talavera Road, Sydney, NSW, 2109, Australia, 61 2 9850 2403

**Keywords:** artificial intelligence, AI, digital scribes, medical scribes, electronic documentation, patient safety, error prevention

## Abstract

Artificial intelligence (AI) scribes, software that can convert speech into concise clinical documents, have achieved remarkable clinical adoption at a pace rarely seen for digital technologies in health care. The reasons for this are understandable: the technology works well enough, it addresses a genuine pain point for clinicians, and it has largely sidestepped regulatory requirements. In many ways, clinical adoption of AI scribes has also occurred well ahead of robust evidence of their safety and efficacy. The papers in this theme issue demonstrate real progress in the technology and evidence of its benefit: documentation times are reported to decrease when using scribes, clinicians report feeling less burdened, and the notes produced are often of reasonable quality. Yet as we survey the emerging evidence base, there remains one outstanding and urgent unanswered question: Are AI scribes safe? We need to know the clinical outcomes achievable when scribes are used compared to other forms of note taking.

## Beyond Efficiency

Early evaluation of any technology naturally focuses on its primary promise, in the case of scribes [1], reducing documentation burden. The studies in this collection confirm that artificial intelligence (AI) scribes deliver on this front. Kanaparthy and colleagues’ [2] rapid review found a general trend toward reduced self-reported documentation time and improvements in clinician satisfaction. The comparative analysis by Ha and colleagues [3] demonstrates that current commercial systems can generate reasonable-quality SOAP (subjective, objective, assessment, and plan) notes in about a minute after a 15-minute encounter. These are meaningful findings. Having shown that scribes likely save time in the settings they have so far been evaluated in (eg, primary care and outpatient settings), we can turn to harder questions of safety, clinical reasoning, and wider system-level effects.

## Toward Systematic Harm Measurement

How safe are clinical scribes? Do they make errors, what type of errors might see when using digital scribes, and are these errors clinically consequential? What are the causes of errors, and what then are the harm mitigations we need to put into place?

Several papers in this collection begin this work. Ha and colleagues [3] highlight that none of the systems they evaluated are error free. Biro and colleagues [4] have developed and validated an instrument specifically designed to assess the accuracy and safety of AI scribe outputs, an essential foundation since, without standardized measurement, we cannot compare across systems or track changes over time. Their early work confirms that AI scribes do make errors, and some have patient safety implications.

Digital technologies have the capacity to both reduce human error and generate new error classes [5]. This raises another question worthy of further investigation: Are digital scribe errors equivalent to human errors, or do they have different risk profiles? A human might lose attention momentarily; a digital scribe will not fatigue but may misrecognize words through speech recognition errors. An AI might confidently fabricate a medication or symptom that was never mentioned or omit clinically significant details in the pursuit of conciseness, each reshaping the clinical narrative in different ways.

Equally, we should recognize that the status quo carries its own safety risks: clinician burnout and cognitive overload contribute to errors that scribes may help reduce. A complete safety evaluation must weigh new risks introduced by AI against existing risks that the technology may mitigate and consider what analogous error-detection and correction mechanisms we need to build for AI-generated documentation.

## Clinical Reasoning When Using a Digital Scribe: An Underexplored Frontier

Perhaps the richest opportunity for future research relates to the quality of clinical care when documentation is outsourced to AI. Note-taking is not merely administrative work. When a clinician summarizes their thoughts into a document, they are actively processing information, prioritizing what matters, and forming and testing hypotheses in real time. The clinical note is not just a record of the consultation; it is a cognitive artifact that supports clinical reasoning [6].

What happens to this human sense-making process when documentation is delegated to an AI? We may be freeing the attention of clinicians to be more present with patients, or we may be altering how they would normally think. The Y-KNOT (Your-Knowledgeable Navigator of Treatment) implementation study offers an intriguing signal: expert ratings suggested that while most AI-generated drafts were rated positively, around 1 in 6 preanesthetic assessments were judged to have a negative impact on clinical decision-making [7]. As we document faster, are we also documenting differently? Does that difference matter for patient care? If so, can we mitigate the potential for harm, for example, through clinical training or redesigning the user interaction with a scribe to bring clinicians back into the document-and-reason loop?

## Ecosystem Perspectives

AI scribes sit at a critical position within a clinical workflow. They determine what gets recorded and how it is structured, which in turn shapes what downstream systems, including other AI tools, will see and act on. In this sense, scribes are a gateway technology, creating data layers that propagate through the health system. As we have argued elsewhere in the context of generative AI more broadly, it is helpful to view these technologies through an ecosystem lens that emphasizes system-level properties over isolated components [8]. With this perspective, we can evaluate the broader scribe ecosystem against system-level dimensions: resilience (how does care adapt when the scribe fails?), sustainability (what happens when cloud-based systems change or disappear?), and service interactions (does optimizing documentation affect other aspects of care?).

The patients in Leiserowitz and colleagues’ [9] survey were generally open to an AI scribe when it was framed as supporting clinician focus. Their study also showed one interaction worth monitoring: a meaningful proportion of patients indicated they might withhold sensitive information if an always-listening device was present. Understanding these broader system effects will require looking beyond the scribe itself to the clinical environment it inhabits.

## Regulatory Evolution

There are several reasons why we still lack safety evidence. AI scribes have proliferated [10] in part because they sit outside traditional medical device classification [11]. Many commercial scribes skirt the software as a medical device definition of a decision support system and so have evaded regulation. With no regulatory demands for robust safety evaluation, there is little commercial value, it seems, in publishing commercial safety data. The evidence in this collection suggests that we may need new regulatory thinking, not because current systems are demonstrably unsafe, but because they are different. These systems do influence clinical decisions, and unlike traditional medical devices, they are not static; the large language model underlying a scribe may be updated or retrained over time. Regulatory frameworks designed for deterministic, frozen technologies may need to evolve alongside the technology itself. The question is not whether to regulate but how to do so in ways that preserve innovation while ensuring ongoing safety. Potential mechanisms might include postmarket surveillance requirements, mandatory incident reporting for generative medical AI, or periodic re-evaluation as underlying models are updated.

## Conclusion

The papers in this theme issue represent important progress. AI scribes appear to deliver on their core promise of reducing documentation burden, and the field is developing increasingly rigorous evaluation approaches. Building on this foundation, we see an opportunity to expand what we measure: systematic assessment of errors and harms, investigation of effects on clinical reasoning, and attention to ecosystem-level dynamics. Documentation burden is real, and technologies that address it are welcome. The next chapter of research can help ensure that the time saved translates into better care.

## Abbreviations

AI: artificial intelligence
SOAP: subjective, objective, assessment, and plan
Y-KNOT: Your-Knowledgeable Navigator of Treatment
